# Supplementary figures and images for: Inference of Ancestral Recombination Graphs through Topological Data Analysis
Source: PLoS Comput Biol. 2016 Aug 17;12(8):e1005071. doi: 10.1371/journal.pcbi.1005071 (PMC4988722; doi:10.1371/journal.pcbi.1005071)

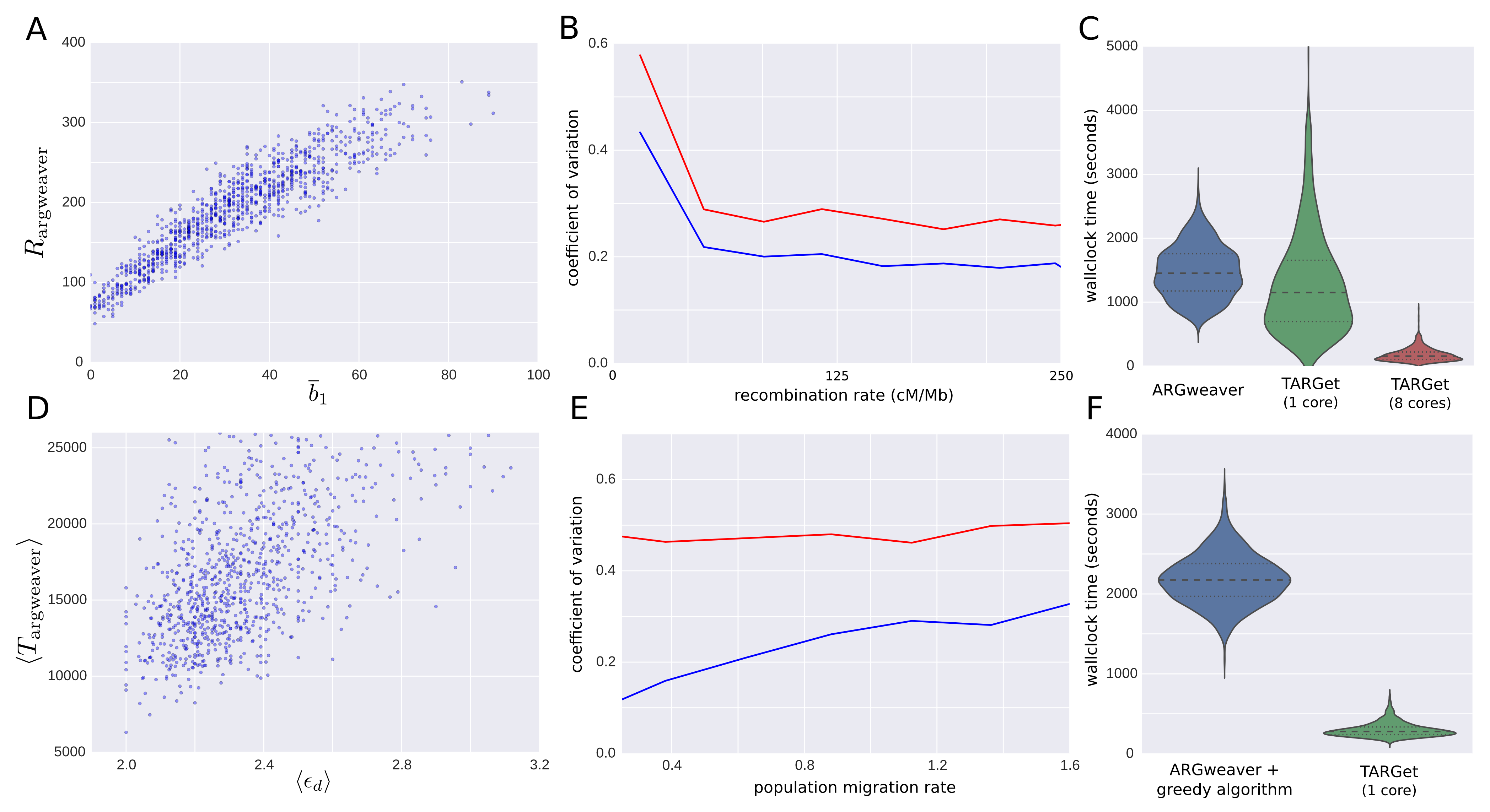

Supplement: S1 Fig — (A) The number of recombination events in SMC ARGs [34], Rargweaver, plotted against the number of bars in the barcode ensemble, b¯1. Both quantities are strongly correlated (Pearson’s r = 0.93, p < 10−100). Plot based on 1,000 coalescent model simulations of a sample of 200 sequences. (B) Coefficient of variation of Rargweaver (blue) and b¯1 (red) as a function of the recombination rate. (C) Distribution of wall-clock running times for the simulations in (A). (D) Average time to the most recent common ancestor of recombining sequences in SMC ARGs, 〈Targweaver〉, plotted against the average death time of bars in the barcode ensemble, 〈ϵd〉, for two divergent populations with recombination and migration. Both quantities are largely correlated (Pearson’s r = 0.55, p < 10−72). Plot based on 900 simulations of a sample of 150 sequences. (E) Coefficient of variation of 〈Targweaver〉 (blue) and 〈ϵd〉 (red) as a function of the migration rate. (F) Distribution of wall-clock running times for the simulations in (D). ARGweaver running times also include the time required to extract 〈Targweaver〉 from SMC ARGs. (TIFF) [file pcbi.1005071.s001.tiff]
